# PP1-Dependent Formin Bnr1 Dephosphorylation and Delocalization from a Cell Division Site

**DOI:** 10.1371/journal.pone.0146941

**Published:** 2016-01-15

**Authors:** Minami Orii, Keiko Kono, Hsin-I Wen, Makoto Nakanishi

**Affiliations:** Department of Cell Biology, Graduate School of Medical Sciences, Nagoya City University, 1 Kawasumi, Mizuho-cho, Mizuho-ku, Nagoya 467–8601, Japan; Hiroshima Universtiy, JAPAN

## Abstract

Cell cycle ends with cytokinesis that is the physical separation of a cell into two daughter cells. For faithful cytokinesis, cells integrate multiple processes, such as actomyosin ring formation, contraction and plasma membrane closure, into coherent responses. Linear actin assembly by formins is essential for formation and maintenance of actomyosin ring. Although budding yeast’s two formins, Bni1 and Bnr1, are known to switch their subcellular localization at the division site prior to cytokinesis, the underlying mechanisms were not completely understood. Here, we provide evidence showing that Bnr1 is dephosphorylated concomitant with its release from the division site. Impaired PP1/Glc7 activity delayed Bnr1 release and dephosphorylation, Bni1 recruitment and actomyosin ring formation at the division site. These results suggest the involvement of Glc7 in this regulation. Further, we identified Ref2 as the PP1 regulatory subunit responsible for this regulation. Taken together, Glc7 and Ref2 may have a role in actomyosin ring formation by modulating the localization of formins during cytokinesis.

## Introduction

Cytokinesis is the final stage of cell cycle, which equally distributes cellular content into two daughter cells. To achieve faithful cytokinesis, cells are equipped with elaborate cell division mechanisms. Actomyosin-dependent formation of contractile ring is critical for successful cytokinesis in diverse eukaryotes. Cdc5/Polo kinase triggers activation of Rho1/RhoA at the division site in anaphase, leading to recruitment and activation of linear actin nucleator formin 1,2]. Accumulating evidence suggest that loss of cell polarity before entering cytokinesis is also critical for actomyosin ring formation, which is regulated at least in part by inhibition of Rac- and PAK1-dependent adhesion in mammals [3,4], or by inhibition of Cdc42 in yeast [5].

The formin family proteins promote linear actin nucleation and elongation through barbed-end binding [6]. Diaphanous-related formins (DRFs) are auto-inhibited by the binding between N-terminal Diaphanous inhibitory domain (DID) and C-terminal Diaphanous autoregulatory domain (DAD). This auto-inhibition could be relieved primarily by the binding of DRFs to Rho type small GTPases [6]. Moreover, phosphorylation plays key roles in formin activation and its proper localization [7].

The budding yeast *S*. *cerevisiae* is a genetically tractable model organism in which regulatory mechanisms of actin nucleation by formins and their physiological significance have been elucidated. Unlike mammalian cells that contain more than fifteen formin isoforms, a budding yeast conceives only two formins, Bni1 and Bnr1 [8,9]. From G_1_/S to metaphase, Bni1 localizes at the growth point in the daughter cell (tip of the bud), whereas Bnr1 localizes at the pre-determined division site, namely bud neck [10]. Bnr1 localization at the bud neck requires Elm1 kinase and MARK/Par1-related kinase Gin4 [11]. In anaphase, Bnr1 disappeared and then Bni1 is recruited at the division site [12,13]. Global analysis identified Cdc14 phosphatase which plays a role in Bnr1 release and Bni1 recruitment [13]. However, it was not clear whether Bnr1 undergoes dephosphorylation concomitant with its release from the division site.

PP1 is a well-characterized phosphatase regulating various cell cycle processes such as mitotic progression [14]. Analogous to higher eukaryotes counterpart, budding yeast PP1/Glc7 is regulated by a large family of regulatory subunits [15,16]. PP1 is a stable protein and its activity is relatively constant during the course of cell cycle progression [17], but its spatial and temporal functions are specified by its regulatory subunits.

Here, we show that Bnr1 is indeed dephosphorylated concomitant with its release from the division site. We further propose that PP1/Glc7 and its regulatory subunit Ref2 may play a role in the release and dephosphorylation of Bnr1, Bni1 recruitment and actomyosin ring formation.

## Results

### Bnr1 is dephosphorylated concomitant with its release from the division site

**Fig 1:**
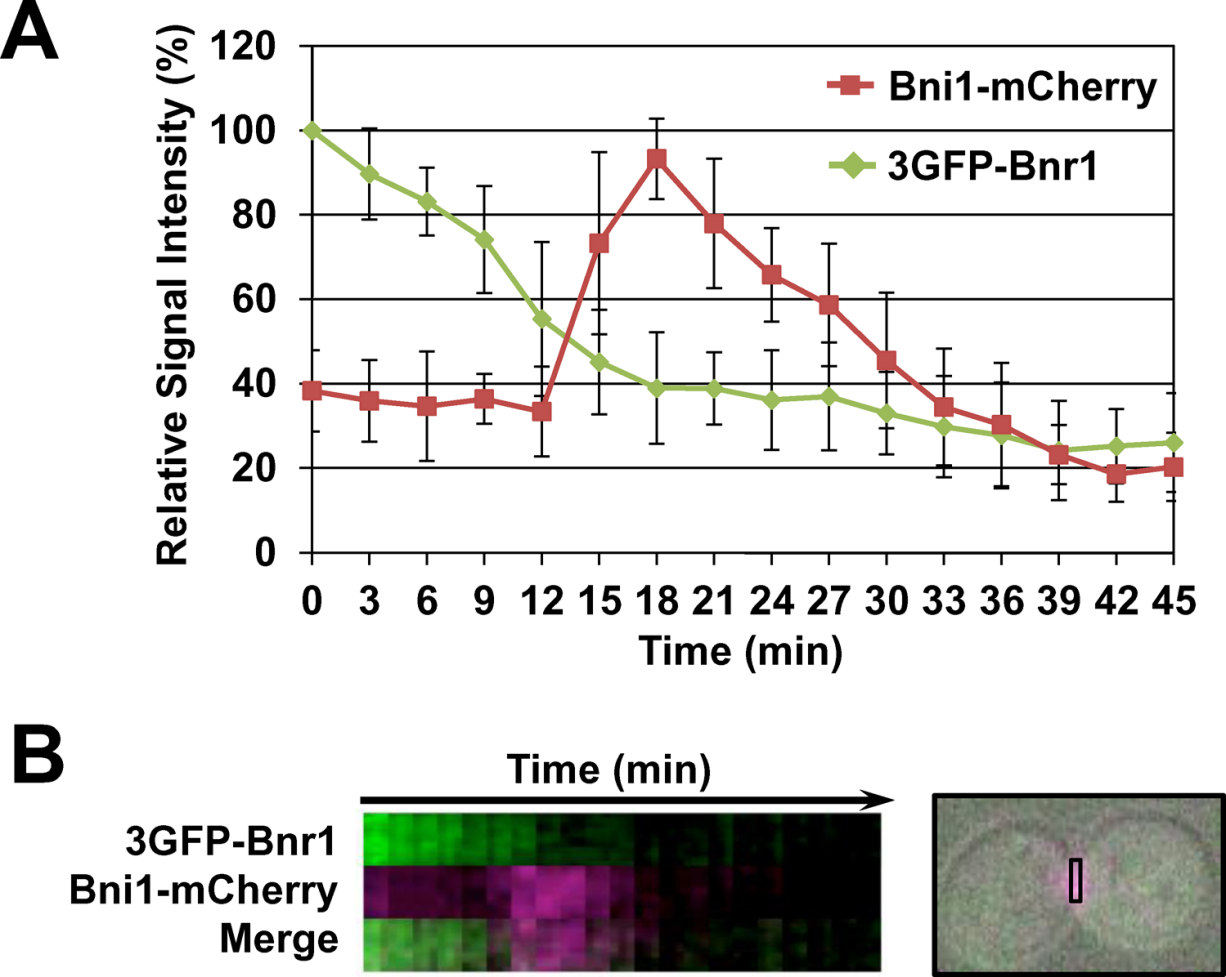
Live-cell imaging for 3GFP-Bnr1 and Bni1-mCherry. Cells expressing 3GFP-Bnr1 and Bni1-mCherry from own promoter were cultured to log phase, spotted on SD medium containing 1.2% agarose on glass slides. Fluorescent signals were monitored under the microscope. (A) Mean relative signal intensities of 3GFP-Bnr1 and Bni1-mCherry at the bud neck were shown. N = 11. Error bars indicate SD. (B) Left, a representative kymograph of (A) is shown. Right, a rectangle indicates an area shown in left panels.

**Fig 2:**
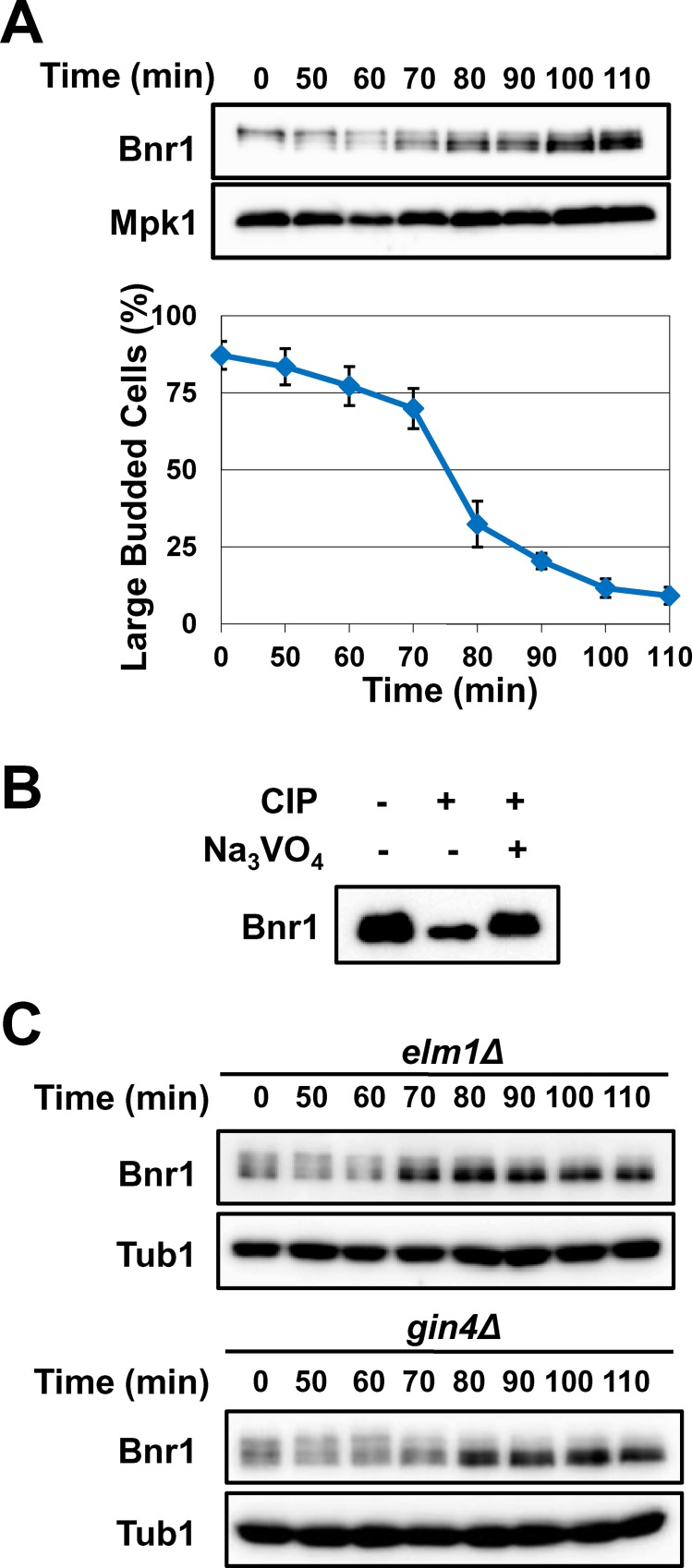
Bnr1 is dephosphorylated during cytokinesis. A, in mitosis by nocodazole and released into fresh media. Samples were collected at indicated time points. Cell lysates were prepared and were subjected to western blotting using anti-myc antibody and anti-Tub1 antibody (control). % cells with large buds in the bright-field microscopic images were counted after formaldehyde fixation to monitor cytokinesis. N>200 for each experiment. Means of three independent experiments are shown. Error bars indicate SD of three independent experiments. B, Bnr1-13myc was immunoprecipitated from cell lysate prepared from asynchronous yeast culture using anti-myc antibody. Beads-bound Bnr1-13myc was subjected to CIP treatment at 37°C in the presence or absence of phosphatase inhibitor Na_3_VO_4_. Samples were subjected to western blotting. Bnr1-13myc signals were detected using anti-myc antibody. C, Cells lacking Bnr1 kinases were synchronized and released as in A, and were subjected to western blotting using anti-myc antibody and anti-Tub1 antibody (control).

To examine subcellular localization of budding yeast’s two formins by live cell imaging, we constructed a yeast strain co-expressing Bni1-mCherry and 3GFP-Bnr1 under regulation by their own promoters. Bnr1 release from the division site was initiated before the peak of Bni1 recruitment (Fig 1A and 1B), consistent with the previous report utilizing synchronized culture [13]. To investigate whether Bnr1 is dephosphorylated concomitant with its release, we next examined posttranslational modifications of Bnr1. After synchronization at metaphase by nocodazole treatment, fast-migrating signals of Bnr1 were detected at 70 min after release, prior to cell separation (Fig 2A). The rapid migration was likely due to Bnr1 dephosphorylation because treatment of the extracts with calf intestinal phosphatase (CIP) resulted in the similar mobility shift of the Bnr1 bands (Fig 2B). These results suggest that Bnr1 is dephosphorylated during cytokinesis. Deletion of *GIN4* or *ELM1*, kinases responsible for Bnr1 phosphorylation[11], indeed increased the potion of fast-migrating signals, further supporting that Bnr1 is dephosphorylated during cytokinesis (Fig 2C).

### PP1/Glc7 is required for proper timing of actomyosin ring formation

**Fig 3:**
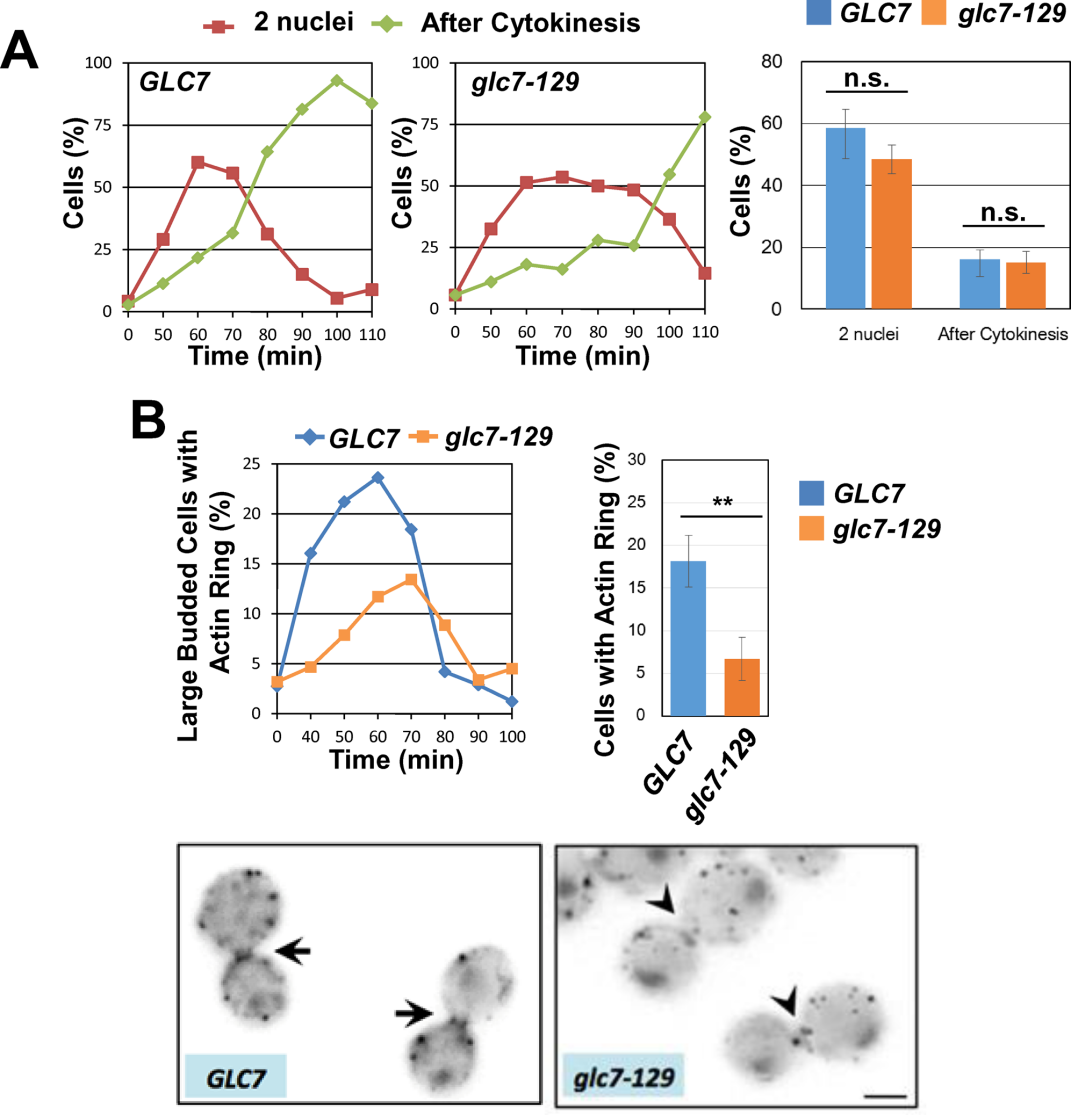
Glc7 is involved in cytokinesis. A, *glc7-129* cells and isogenic control cells were synchronized and released at 25°C (permissive temperature) as in Fig 2. Mitosis and cytokinesis were monitored after formaldehyde fixation by DAPI staining and cell separation, respectively. (Left and center) Representative results of three independent experiments were shown. (Right) % cells after nuclear division (2 nuclei) and % cells after cytokinesis (After Cytokinesis) at 60 min after release were shown. N>150 for each experiment. Means of three independent experiments. Error bars, s.e.m. n.s. (not significant), statistically insignificant by student’s t-test. B, *glc7-129* cells and isogenic control cells were synchronized and released at 25°C (permissive temperature) as in Fig 2. Large budded cells with actin ring was counted after formaldehyde fixation and Allexa-phalloidin staining. (Upper left) Representative results of three independent experiments were shown. (Upper right) % cells with actin ring at 60 min after release were shown. N>150 for each experiment. Means of three independent experiments. Error bars, s.e.m. **; p<0.01 by student’s t-test. (Lower) Arrows indicate actin ring (*GLC7*), arrow heads indicate absence of actin ring (*glc7-129*). A scale bar, 2μm.

It is known that Cdc14 is involved in Bnr1 release [13]. We examined the possibility whether other phosphatase could also be involved in this regulation. Although PP1/Glc7 is a well-established mitosis (karyokinesis) regulator, other lines of evidence suggest that Glc7 has a specific role in actomyosin ring formation: Glc7 localizes to the division site, is involved in cell separation, and a *glc7* mutant allele have the actin depolarization defect [18–20]. To examine the possibility, we cultured *glc7-129* cells at permissive temperature. As previously described, at the permissive temperature, *glc7-129* cells underwent relatively normal nuclear division, when monitored by DAPI staining (Fig 3A)[19]. In this condition, we found significant delay of actomyosin ring formation in *glc7-129* cells (Fig 3B). These results are consistent with the hypothesis that Glc7 may have role in actomyosin ring formation.

### PP1/Glc7 is involved in Bnr1 dephosphorylation, release and recruitment of Bni1 at the division site

**Fig 4:**
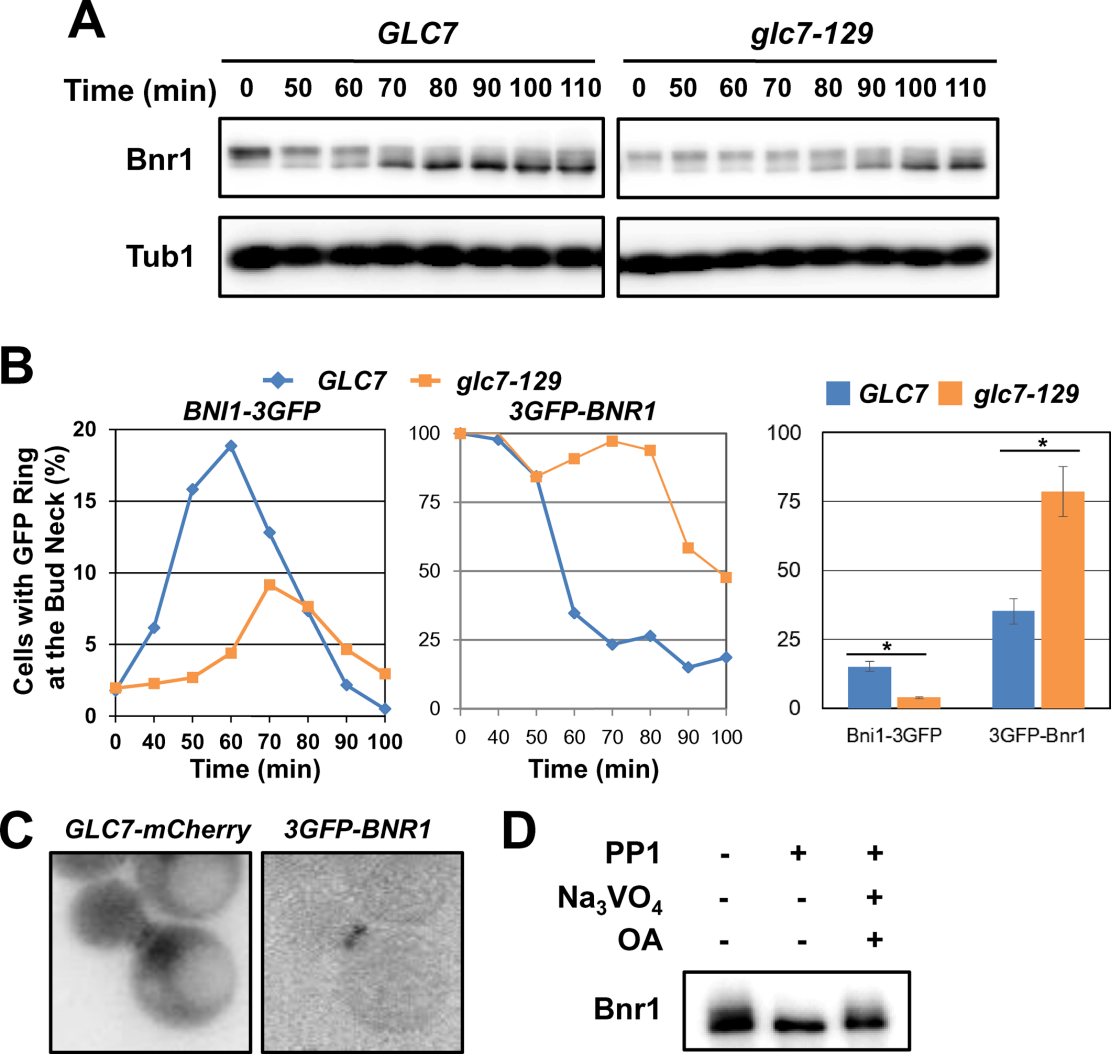
Glc7 is involved in Bnr1 release and dephosphorylation. A, *glc7-129* cells and isogenic control cells expressing Bnr1-13myc from own promoter were synchronized, released and analyzed as in Fig 2A. B, *glc7-129* cells and isogenic control cells expressing *BNI1-3GFP* or *3GFP-BNR1* from own promoters were synchronized, released as in Fig 2A. Cells were fixed with formaldehyde at 25°C for 5 min, and then GFP signal forming a single ring at the division site was counted under the fluorescent microscopy. (Left) Representative results of three independent experiments were shown. (Right) % cells with single ring Bni1-3GFP signal (Bni1-3GFP) and % cells with 3GFP-Bnr1 signal (3GFP-Bnr1) at 60 min after release were shown. N>200 for each experiment. Means of three independent experiments were shown. Error bars, s.e.m. *; p<0.05 by student’s t-test. C, Cells expressing *GLC7-mCherry* and *3GFP-BNR1* from own promoters were observed under the fluorescent microscopy. D, Bnr1-13myc was immunoprecipitated from cell lysate prepared from asynchronous yeast culture using anti-myc antibody. PP1 treatment was performed at 30°C for 30 min in the presence or absence of phosphatase inhibitors Na_3_VO_4_ and Okadaic Acid (OA).

To determine the involvement of Glc7 in Bnr1 dephosphorylation, *glc7-129* and control cells were synchronized at metaphase by nocodazole treatment, released, and then the kinetics of Bnr1 dephosphorylation was examined. Bnr1 dephosphorylation was delayed in *glc7-129* cells (Fig 4A), suggesting that Glc7 may play a role in the dephosphorylation of Bnr1, either directly or indirectly. Both Bnr1 release and Bni1 recruitment from the division site were impaired in *glc7-129* cells (Fig 4B). We also observed the colocalization of Bnr1-3GFP and Glc7-mCherry at the division site (Fig 4C). *In vitro* dephosphorylation assay demonstrated that mammalian homologue of Glc7(PP1) was capable of dephosphorylating Bnr1 (Fig 4D). Although we do not exclude critical regulation by other phosphatases such as Cdc14, one possible explanation of our results are Glc7 promotes dephosphorylation and/or release of Bnr1, Bni1 recruitment and actomyosin ring formation.

### Ref2 is a PP1 regulatory subunit required for actomyosin ring formation

**Fig 5:**
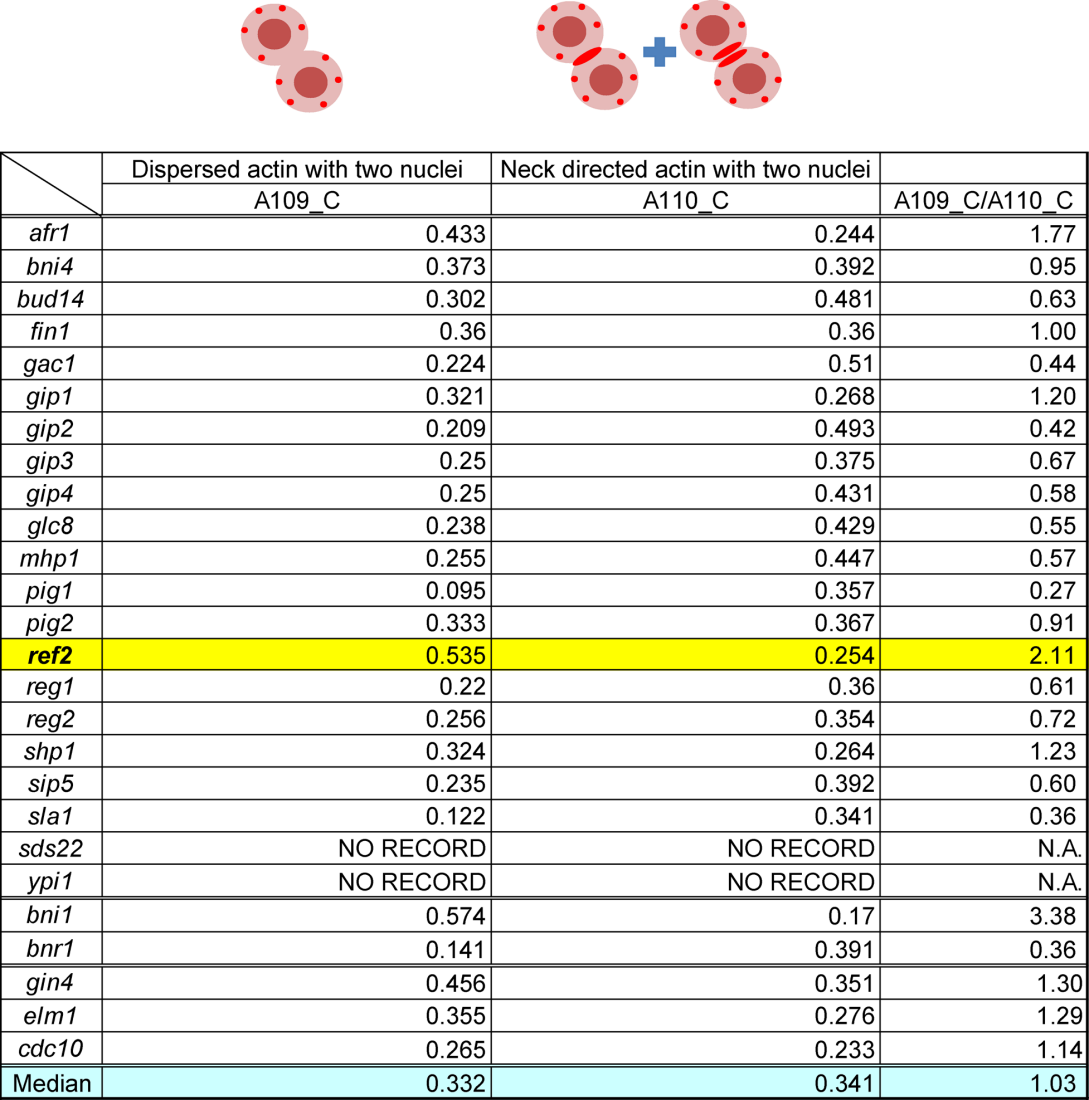
In silico screening identifies PP1 regulatory subunit Ref2 as a potential cytokinesis regulator. Saccharomyces cerevisiae morphological database [21] was used for in silico screening. We focused on two parameters, dispersed actin with divided nuclei (A109_C) and directed actin at the bud neck with divided nuclei (A110_C). Median of all mutant strains analyzed in the database were shown as a control value. Deletion mutants for Glc7 regulatory subunits (from *afr1Δ* to *ypi1Δ*), formins (*bni1Δ* and *bnr1Δ*) septin related kinases (*gin4Δ* and *elm1Δ*) and a septin (*cdc10Δ*) were analyzed. N.A., not assigned.

Substrate specificity of PP1/Glc7 is dictated by the regulatory subunits that direct its catalytic activity toward specific substrate(s) [15,16]. Therefore we screened PP1 regulatory subunits to identify factor(s) required for cytokinetic functions through modulating PP1/Glc7 activity. We performed *in silico* screening utilizing SCMD database [21], where budding yeast mutant traits were comprehensively and quantitatively analyzed [21]. To evaluate cytokinetic defects, we examined two parameters in the database: “dispersed actin with two nuclei (the parameter A109_C in the database)” and “neck-directed actin with two nuclei (A110_C)”. By comparing these two parameters, *ref2Δ* cells were suggested to show significant defect in actin organization during cytokinesis (Fig 5). This phenotype was analogous to that of *bni1Δ* but not *bnr1Δ* (Fig 5.), further supporting Ref2’s involvement in negative regulation of Bnr1. In septin/ septin related kinase-defective cells (*gin4Δ*, *elm1Δ* and *cdc10Δ*), the actin defects have similar tendency but relatively milder than the defect of *ref2Δ* cells. Thus, Ref2 is potentially involved in actin organization during cytokinesis, either directly or indirectly via septin regulation.

**Fig 6:**
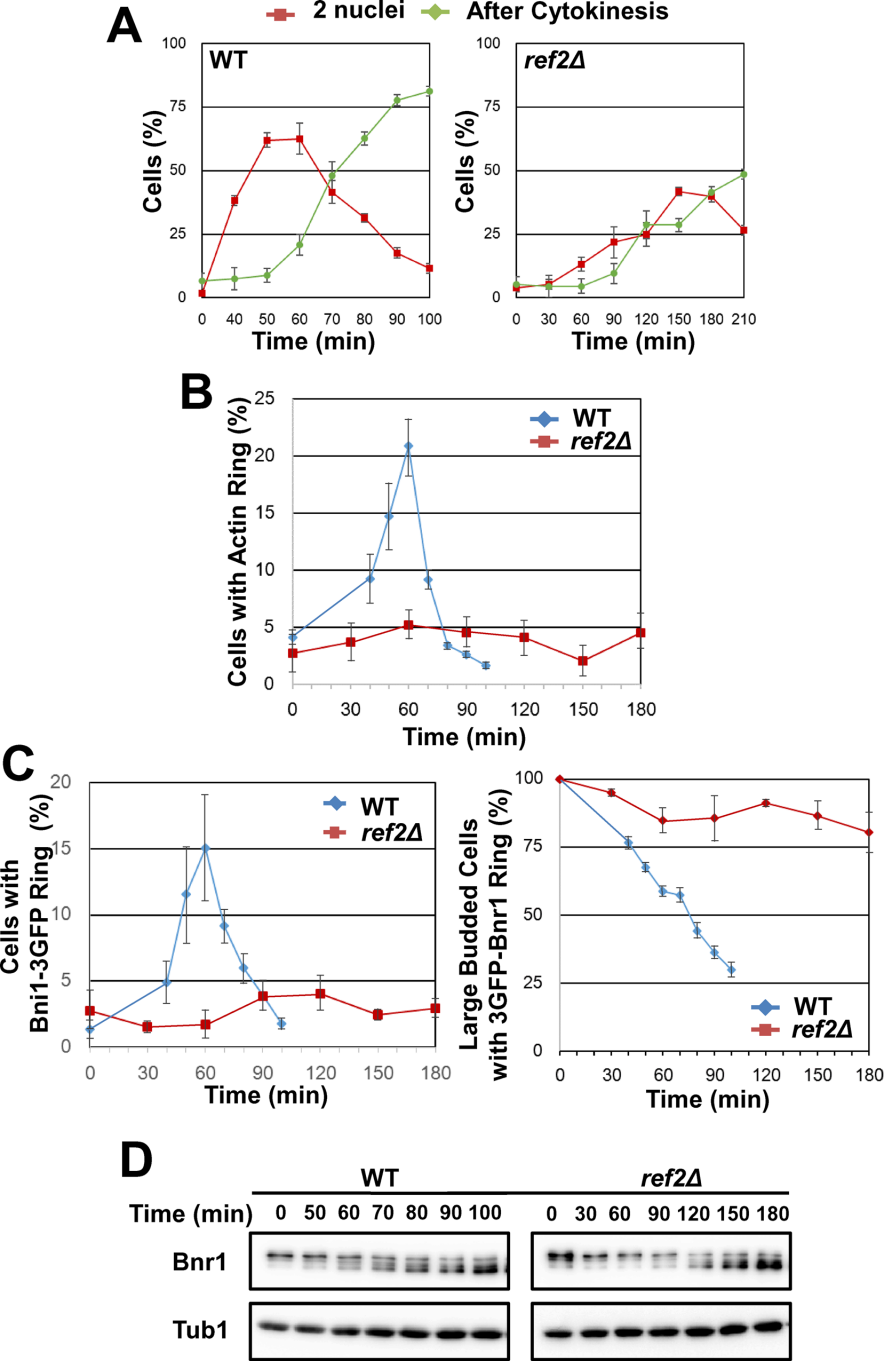
Ref2 is required for formin switching and actomyosin ring formation. A, *ref2Δ* cells and isogenic control cells were synchronized, released and analyzed as in Fig 3. Note that in this and subsequent figures, time points for the observation were not identical between *ref2Δ* and control cells, due to slow growth phenotype of *ref2Δ* cells. N>100 for each experiment. Means of three independent experiments were shown. Error bars, s.e.m. B, *ref2Δ* cells and isogenic control cells were synchronized, released and analyzed as in Fig 3. N>100 for each experiment. Means of three independent experiments were shown. Error bars, s.e.m. C, *ref2Δ* cells expressing *BNI1-3GFP* or *3GFP-BNR1* from own promoters were synchronized, released and analyzed as in Fig 3. N>100 for each experiment. Means of three independent experiments were shown. Error bars, s.e.m. D, *ref2Δ* cells expressing Bnr1-13myc were synchronized, released and analyzed as in Fig 2A.

To further confirm Ref2’s role in cytokinesis, *ref2Δ* cells were synchronized at metaphase by nocodazole and then released into anaphase to examine the formation of actomyosin ring. Actomyosin ring formation was markedly impaired in *ref2Δ* cells (Fig 6A and 6B). In addition, both Bnr1 release and Bni1 recruitment were severely impaired in *ref2Δ* cells (Fig 6C). Further, Bnr1 dephosphorylation was delayed in *ref2Δ* cells (Fig 6D). Altogether, Glc7 and Ref2 is involved in Bnr1 dephosphorylation and/or release.

**Fig 7:**
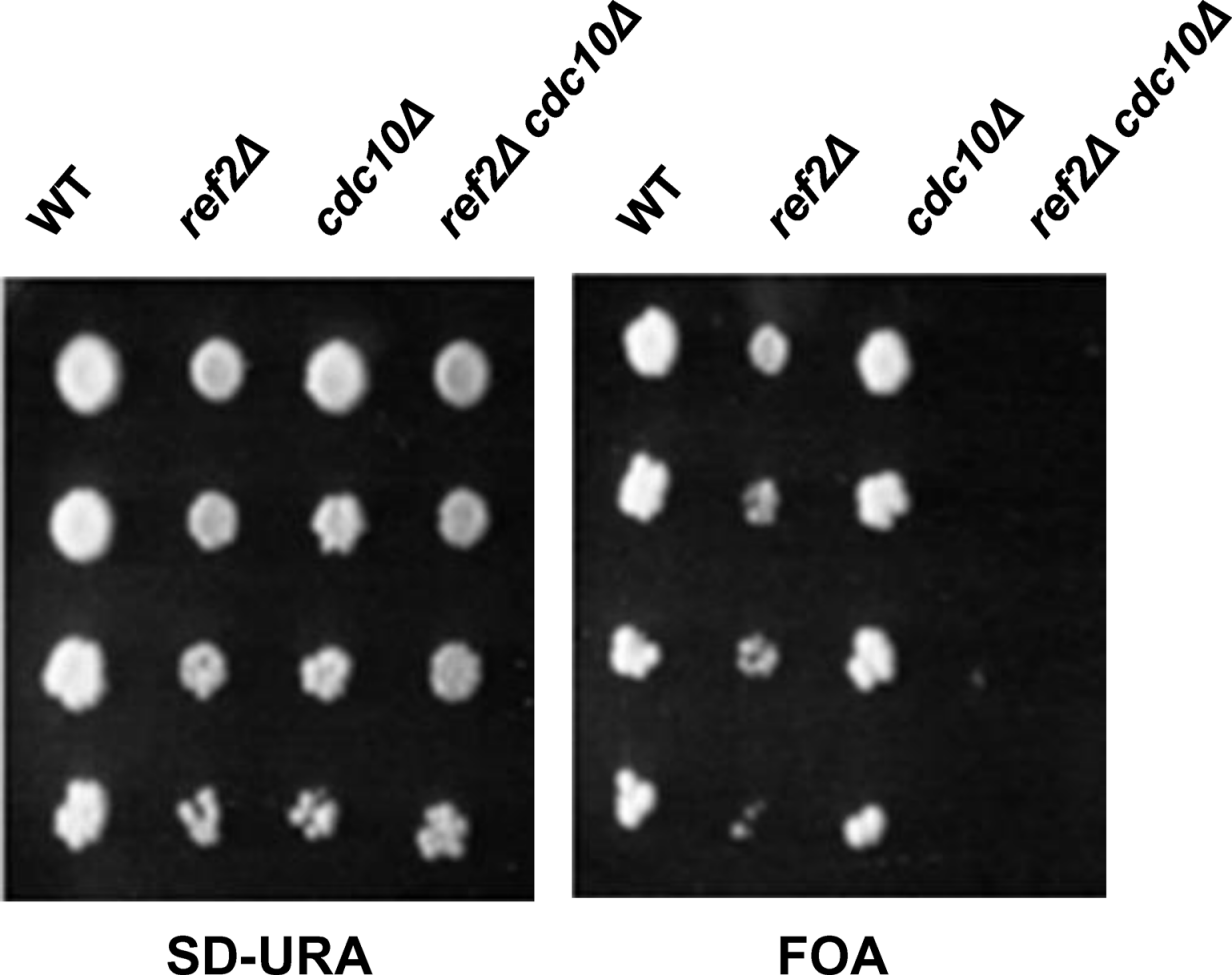
Genetic evidence supporting *REF2*’s involvement in cytokinesis. Indicated strains with a plasmid (pRS316-*CDC10*) were serially diluted (4X), spotted onto FOA (right) or SD-URA (left) plates and incubated at 25°C for 4 days.

A septin mutant *cdc10Δ* is known to show a synthetic growth defect when it is combined with a mutation in pathways involved in actomyosin ring formation [22]. Based on this result, we examined the genetic interaction between *CDC10* and *REF2*. *cdc10Δ*, *ref2Δ* cells were not viable thus demonstrating a clear synthetic lethality between both gene deletions (Fig 7). Although this result is consistent with our model, it should be noted that these results are also consistent with the interpretation that Ref2 regulates septins. Altogether, we propose that Glc7/Ref2 promotes Bnr1 dephosphorylation and/or release from the division site, either directly or indirectly, enabling Bni1 recruitment and actomyosin ring formation.

## Discussion

In this study, we provide evidence that Bnr1 is dephosphorylated during cytokinesis, concomitant with Bnr1 release from the division site. Our results suggest potential involvement of PP1/Glc7 and its regulatory subunit Ref2 in Bnr1 dephosphorylation and/or release from the division site, Bni1 recruitment and actomyosin ring formation.

A major role for Glc7 regulatory subunits is targeting Glc7 to the specific location [15,16]. However, Glc7 localization to the division site in *ref2Δ* cells were comparable to that of control cells (data not shown). Moreover, we confirmed robust localization of Ref2 in nucleus as well as faint signal in cytosol, consistent with the result of the large scale study [23]. Further, Ref2 is critical for transcriptional termination on snoRNA genes and cation homeostasis [24,25]. Therefore, our results do not exclude the possibility that Ref2/Glc7-dependent regulation of Bnr1 could be an indirect consequence of snoRNA or other pathways mediated by Ref2. Further, considering genetic interaction of *ref2Δ* and *cdc10Δ*, it is also possible that Glc7/Ref2-dependent Bnr1 release is mediated by septin regulations.

We showed by live-cell imaging that Bnr1 is gradually released from the division site and Bni1 is recruited to the division site (Fig 1A), consistent with the previous reports [12,13]. Although Bni1 and Bnr1 share an essential function in linear actin filament assembly [8,26–28], actin filaments assembled by each formin have different features. Bni1 assembles thin and short cables, whereas Bnr1 assembles thick and long cables [29]. For the construction of circular structure such as actomyosin ring, thin and short cables are likely to be more suitable. Indeed, loss of Bni1 leads to impaired actin ring formation [10]. Thus, physiological relevance of Bnr1-Bni1 relocalization in anaphase seems to be assembling specific actin filaments appropriate for actomyosin ring formation. We have reported similar Bnr1-Bni1 switching during cell surface damage and repair processes [30]. In this case, Bni1 at the growth point in a daughter cell was degraded and Bnr1 in turn accumulated at the damage site. Therefore, Bni1-Bnr1 switching could be common mechanism for cell polarity changes during cell cycle progression and stress responses.

Although evolutionary conservation of the mechanisms have not examined yet, mammalian PP1γ localizes to cleavage furrow [31], implying that similar mechanism may exist in mammalian cells.

## Materials and Methods

### Media, strains and genetic manipulations

Standard procedures were employed for plasmid constructions, *E*. *coli*, and yeast genetic manipulations. Yeast cells were grown at 25°C in rich (YPD) or synthetic (SD) growth medium supplemented with amino acids. Yeast strains and plasmids are listed in S1 and S2 Tables, respectively.

### Cell synchronization

A yeast culture grown overnight was refreshed and incubated for an additional 2–6 hours until the OD_600_ reached 0.1–0.2. For cell cycle synchronization in mitosis, 15 μg/ml nocodazole was added to the culture for 3–4 hours until large budded cells reach >85%. Although the culture medium was selected appropriately (i.e. cells with URA marker plasmids were grown in SD-URA medium), all the nocodazole treatment was performed in YPD medium to increase the synchronization efficiency. Cells were washed three times and then released to either YPD or SD-URA medium. Samples were collected at indicated time points.

### Microscopy and image analysis

A yeast culture grown overnight was refreshed and incubated for additional 2–6 hours until the OD_600_ reached 0.1–0.3. For live cell imaging, cells were then spotted onto an agarose bed (SD medium+1.2% agarose) on glass slides. Fluorescent images were acquired using an Axio Observer z1 (Carl Zeiss). Image processing and signal quantification was performed using FIJI software. To monitor cell division, cells were fixed at each time point with 4% formaldehyde for seven minutes, washed with PBS for three times and observed under the microscope (bright-field). For DAPI (DNA) and phalloidin (actin) staining, cells were fixed with 4% formaldehyde for 40 minutes at 25°C, washed with PBS for three times. To the 45 μl of cell suspension in PBS, 5 μl of Alexa Fluor 568 phalloidin (molecular probes) and 0.1% of triton X-100 was added, and incubated at 4°C in the dark for 30 minutes. Cells were washed three times with PBS and observed under the microscope. To monitor nuclear division, cells were stained with 1μg/ml DAPI (SIGMA) and immediately observed under the microscope. For 3GFP-Bnr1 and Glc7-mCherry co-localization, cells were fixed with 4% formaldehyde for seven minutes, washed with PBS for three times, and observed under the microscope. As previously described, Bni1-3GFP signals could be detected as a single ring during actomyosin ring formation, as well as double rings during abscission [12]. To specifically analyze Bni1 during actomyosin ring formation, cells with single ring Bni1-3GFP signal was scored. For the analysis of 3GFP-Bnr1, Dumbbell shape cells with 3GFP-Bnr1 cells at time 0 was scored as 100%. For the analysis of 3GFP-Bnr1 in *ref2Δ* cells, the dumbbell shape cells were scored as 100% to specifically describe the 3GFP-Bnr1 localization before cell separation.

### Biochemical methods

For Bnr1-13myc western blotting, a yeast culture grown overnight (OD_600_ ~ 0.1). 2.5OD equivalent of cells were synchronized and released as described above. 0.5–1 OD equivalent of cells were collected at each time point. Proteins were extracted using Trichloroacetic acid (TCA) as follows. Cells were suspended in 0.2M NaOH/β-Mercaptoethanole for 7 min at 4°C, and then 80μl of 50% TCA was added and stand on ice for 7 min. After centrifugation, the pellet was washed once with ice-cold Acetone, pelleted again, dried, and then resuspended in 2X laemmmli buffer for SDS-PAGE. Denaturing was performed at 65°C for 15 min to avoid protein degradation during boiling. For phosphatase treatment, yeast cells expressing Bnr1-13myc from own promoter were pelleted and frozen at -80°C. Pellets were suspended in ice-cold RIPA buffer [50 mM Hepes-KOH (pH7.4), 100 mM KCl, 1mM EDTA, cOmplete protease inhibitor (Sigma), 1mM DTT, 10 mM PMSF], subjected to beads beating using Fastprep (MP biomedicals) at 4°C. Cell lysates were clarified by centrifugation, incubated with c-myc antibody (9E10; SANTA CRUZ) for one hour at 4°C, and then incubated with protein G sepharose (GE healthcare) for one hour at 4°C. Beads-bound Bnr1-13myc were washed three times with RIPA buffer without protease inhibitors, and subjected to the phosphatase treatment using Calf Intestinal (CIP) (NewEngland Biolabs) or rabbit Protein phosphatase 1 purified from *E*. *coli* (NewEngland Biolabs), following manufacturer’s instructions. Each phosphatase treatment was performed in the presence or absence of phosphatase inhibitors as indicated in figures (5 mM Na_3_VO_4_ and 2.5 mM ocadaic acid). Standard procedures were employed for SDS-PAGE and Western blotting. Mouse anti-c-myc antibody (9E10; SANTA CRUZ), rat anti-Tubαantibody (AbD serotec), goat Mpk1 antibody (SANTA CRUZ) and mouse anti-GFP antibody (Roche) were utilized to detect the western blot signals.

### In silico screening

Saccharomyces cerevisiae morphological database [21] was used for in silico screening. We focused on two parameters, dispersed actin with divided nuclei (A109) and directed actin at the bud neck with divided nuclei (A110).

## Supporting Information

S1 Table Yeast strains used in this study. (DOCX)

S2 Table Plasmids used in this study. (DOCX)
