# Supplementary material for: PP1-Dependent Formin Bnr1 Dephosphorylation and Delocalization from a Cell Division Site
Source: PLoS One. 2016 Jan 15;11(1):e0146941. doi: 10.1371/journal.pone.0146941 (PMC4714816; doi:10.1371/journal.pone.0146941)
Supplement: S1 Table — (DOCX) [file pone.0146941.s001.docx]

S1 Table. Yeast strains used in this study.

| Name | Genotype | Source |
| --- | --- | --- |
| YKK12 | *MAT a his3, leu2, met15, ura3 BNR1-13myc-HIS3* | D. Pellman |
| YKK1009 | *MAT a his3, leu2, met15, ura3 Bnr1-13myc gin4::Km* | This study |
| YKK1015 | *MAT a his3, leu2, met15, ura3 Bnr1-13myc elm1::Km* | This study |
| KT1113 | *MAT α leu2 ura3 his3 (WT for glc7-129)* | K. Tatchell |
| KT1622 | *MAT α leu2 ura3 his3 glc7-129* | K. Tatchell |
| YKK1163 | *MAT α GLC7 leu2 ura3 his3 BNR1-13myc:HIS3* | This study |
| YKK1171 | *MAT α glc7-129 leu2 ura3 his3 BNR1-13myc:HIS3* | This study |
| YKK1827 | *MAT a his3, leu2, met15, ura3 ref2::Km BNR1-13myc:HIS3* | This study |
| YKK1720 | *MAT a his3, leu2, met15, ura3 ref2::HIS3* | This study |
| YKK1779 | *MAT a his3, leu2, met15, ura3 ref2::HIS3 BNI1-3GFP:LEU2* | This study |
| YKK1785 | *MAT a his3, leu2, met15, ura3 ref2::HIS3 [pBNR1-3GFP-BNR1]* | This study |
| YKK1759 | *MAT a his3, leu2, met15, ura3 ref2::HIS3 [pCDC10-CDC10]* | This study |
| YKK1818 | *MAT a his3, leu2, met15, ura3 [pCDC10-CDC10]* | This study |
| YKK1821 | *MAT a his3, leu2, met15, ura3 cdc10::Km [pCDC10-CDC10]* | This study |
| YKK2306 | *MAT a his3, leu2, met15, ura3 ref2::HIS3 cdc10::Km [pCDC10-CDC10]* | This study |
| YKK1041 | *MAT α leu2 ura3 his3 glc7-129 [pBNR1-3GFP-BNR1]* | This study |
| YKK1051 | *MAT α leu2 ura3 his3 GLC7 [pBNR1-3GFP-BNR1]* | This study |
| YKK1070 | *MAT α leu2 ura3 his3 glc7-129 BNI1-3GFP:LEU2* | This study |
| YKK1117 | *MAT α leu2 ura3 his3 GLC7 BNI1-3GFP:LEU2* | This study |
| YKK2064 | *MAT a his3, leu2, met15, ura3 BNI1-mCherry:Km[pBNR1-3GFP-BNR1]* | This study |
