# Supplementary material for: PP1-Dependent Formin Bnr1 Dephosphorylation and Delocalization from a Cell Division Site
Source: PLoS One. 2016 Jan 15;11(1):e0146941. doi: 10.1371/journal.pone.0146941 (PMC4714816; doi:10.1371/journal.pone.0146941)
Supplement: S2 Table — (DOCX) [file pone.0146941.s002.docx]

S2 Table. Plasmids used in this study.

| Name | Description | Source |
| --- | --- | --- |
| PB2921 | *pBNR1-3GFP-BNR1*, *URA3*, Cen | D. Pellman |
| pKK395 | *pCDC10*-*CDC10*, *URA3*, Cen | This study |
